# Environmental and Regulatory Control of RTX Toxins in Gram-Negative Pathogens

**DOI:** 10.3390/toxins18010027

**Published:** 2026-01-06

**Authors:** Hossein Jamali, Tylor Pereira, Charles M. Dozois

**Affiliations:** 1 Institut National de la Recherche Scientifique (INRS), Centre Armand-Frappier Santé Biotechnologie, 531 Boul. des Prairies, Laval, QC H7V 1B7, Canada; hossein.jamali@inrs.ca (H.J.); tylor.pereira@inrs.ca (T.P.); 2 Centre de Recherche en Infectiologie Porcine et Avicole (CRIPA), Faculté de Médecine Vétérinaire, Université de Montréal Saint-Hyacinthe, Saint-Hyacinthe, QC J2S 2M2, Canada; 3 Pasteur Network

**Keywords:** RTX toxin regulation, Gram-negative pathogens, virulence gene control, environmental signaling, two-component systems

## Abstract

Repeat-in-toxin (RTX) toxins are calcium-dependent exoproteins secreted by diverse Gram-negative bacteria and play central roles in cytotoxicity, immune modulation, and tissue colonization. While their structure and secretion mechanisms are well-characterized, the regulation of RTX toxin expression remains complex and species-specific. This review provides a comprehensive overview of the regulatory networks governing RTX gene expression, highlighting both conserved mechanisms and niche-specific adaptations. RTX genes are controlled by multilayered regulatory systems that integrate global transcriptional control, metabolic regulation, and environmental sensing. Expression is further shaped by host-derived signals, physical contact with host cells, and population-dependent cues. Quorum sensing, post-transcriptional regulation by small RNAs, and post-translational activation mechanisms contribute additional layers of control to ensure precise regulation of toxin production. We also explore how RTX regulation varies across anatomical niches, including the gut, lung, bloodstream, and biofilms, and how it is co-regulated with broader bacterial virulence. Finally, we discuss emerging insights from omics-based approaches and the potential of anti-virulence strategies targeting RTX regulatory pathways. Together, these topics underscore RTX regulation as a model for adaptive virulence control in bacterial pathogens.

## 1. Introduction

Repeat-in-toxin (RTX) toxins are a family of large, pore-forming cytotoxins that serve as key virulence factors in a wide range of Gram-negative pathogens. They are characterized by C-terminally located glycine- and aspartate-rich tandem repeats that bind calcium ions, which play a central role in secretion and activation [1]. Within the low-calcium environment of the bacterial cytoplasm, these toxins remain largely unstructured, preventing premature folding. Upon secretion into calcium-rich environments—such as certain host niches—RTX toxins bind calcium ions and adopt their active conformation [2]. Once secreted and properly folded, RTX toxins insert into host cell membranes via hydrophobic domains, forming lytic pores or disrupting membrane integrity and signaling [3]. The prototypical RTX toxin, *Escherichia coli* HlyA hemolysin, exemplifies this mechanism by lysing erythrocytes and a broad range of host cells [4]. RTX toxins play key roles in virulence by damaging host tissues, evading immune defenses, and modulating host cell signaling [5]. The evolutionary success of RTX toxins lies in their precise regulation and rapid deployment in response to environmental cues [6].

**Figure 1:**
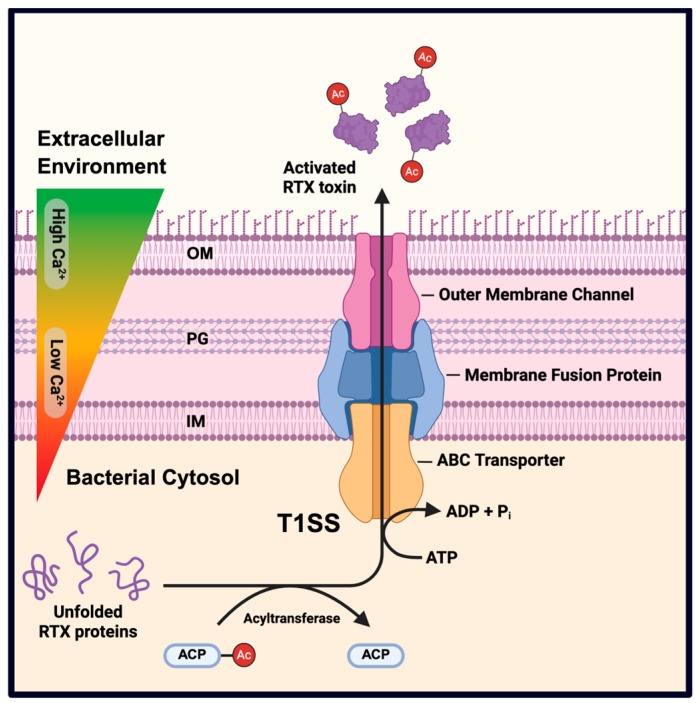
General mechanism of RTX toxin secretion and post-translational activation. RTX toxins are synthesized in the bacterial cytosol as unfolded protoxins and activated by dedicated acyltransferases (e.g., HlyC, CyaC, RtxC), which transfer fatty acyl chains from acyl–acyl carrier protein (ACP) to conserved internal lysine residues. The unfolded, acylated toxin is secreted in a single step across the inner membrane (IM), directly through the periplasmic space, peptidoglycan (PG) layer, and outer membrane (OM) via a type I secretion system (T1SS). The T1SS comprises an ATP-binding cassette (ABC) transporter, a membrane fusion protein, and an outer membrane channel (TolC-like protein). ATP hydrolysis provides the energy required for secretion. Upon release into the extracellular, calcium-rich environment, RTX toxins bind Ca^2+^ through their glycine- and aspartate-rich repeat domains, undergo conformational folding, and acquire their biologically active structure. Created in BioRender. Dozois, C. (2026) https://BioRender.com/oslu16w (accessed on 19 December 2025).

RTX toxins are secreted through a dedicated type I secretion system (T1SS) that transports the unfolded toxin across both the inner and outer membranes in a single step, bypassing the periplasm [1,5]. The T1SS complex typically includes an ATP-binding cassette transporter, a membrane fusion protein, and an outer membrane channel, as exemplified by HlyB, HlyD, and TolC in *E. coli*. This secretion pathway minimizes periplasmic toxicity and allows rapid delivery of virulence factors upon host contact [7,8]. In addition, RTX operons often encode an acyltransferase enzyme, sometimes referred to as an activator protein, which post-translationally modifies the RTX toxin by attaching fatty acyl chains to specific lysine residues. This modification is essential for full cytolytic activity. For instance, HlyA in *E. coli* requires HlyC-mediated acylation, and CyaA in *Bordetella pertussis* is similarly activated by CyaC [9]. Together, the structural modularity, efficient secretion, and post-translational activation of RTX toxins reflect their evolutionary adaptation as potent bacterial virulence factors [2,9]. An overview of RTX toxin secretion, post-translational acylation, and calcium-dependent activation is shown in Figure 1.

In this review, we present current knowledge on the regulation of RTX toxin expression across diverse bacterial pathogens. We discuss the environmental, genetic, and host-derived signals that govern RTX operon activity, highlighting both well-characterized regulators and emerging pathways. Special attention is given to niche-specific modulation, experimental models, and the translational potential of targeting RTX regulation for anti-virulence therapy. By identifying conserved mechanisms and unresolved questions, we aim to guide future investigations into how bacteria precisely control the expression of these potent toxins during infection. Because RTX toxins are regulated and critical for virulence but not essential for bacterial viability, their regulatory pathways represent attractive targets for anti-virulence strategies aimed at attenuating pathogens during infection without imposing strong selective pressure for development of resistance.

## 2. Diversity of RTX Toxins Across Bacterial Genera

RTX toxins are widespread among Gram-negative pathogens, with varied biological activities and host specificities [1]. Despite their conserved secretion mechanism and calcium-binding repeats motifs, RTX family members can have different target cells and effects on their function:

### 2.1. Enterobacteriaceae

*Escherichia coli* produces the archetypal RTX hemolysin HlyA, which is widely expressed by uropathogenic (UPEC) and extraintestinal pathogenic strains and functions as a potent cytotoxin capable of lysing erythrocytes and damaging host tissues [4,10]. HlyA lyses red blood cells and other host cells, contributing to tissue damage in urinary tract and sepsis infection models [11]. Some pathogenic *E. coli* carry a plasmid-encoded RTX toxin called EHEC hemolysin (EHEC-Hly, syn. Ehx), similar in function to HlyA [12]. In *E. coli*, the pore-forming RTX toxin HlyA is encoded by the *hlyCABD* gene cluster (HlyA toxin, HlyC activator, HlyB/D secretion components), which can be encoded on either pathogenicity islands within the chromosome or on large virulence plasmids in uropathogenic and other extraintestinal pathogenic strains [13]. HlyA remains one of the best-characterized RTX toxins and serves as a model for understanding RTX gene regulation, secretion, and host cell interaction [4]. Similarly, *Proteus mirabilis* produces HpmA, a pore-forming hemolysin with RTX-like features that mediates host cell damage and cytotoxicity during urinary tract infection [14].

### 2.2. Pasteurellaceae

These veterinary pathogens produce RTX leukotoxins that specifically target immune cells. *Actinobacillus pleuropneumoniae*, the causative agent of porcine pleuropneumonia, secretes multiple RTX toxins—ApxI, ApxII, ApxIII, and ApxIV—that damage lung tissue, neutrophils, and macrophages, leading to hemorrhagic pneumonia [15]. *Mannheimia haemolytica*, a major bovine respiratory pathogen, produces leukotoxin A (LktA), an RTX toxin that specifically lyses ruminant leukocytes by binding CD18 integrins [16]. These toxins are major virulence factors in livestock respiratory infections. RTX encoding operons in these bacteria typically include genes encoding a dedicated T1SS (e.g., the *lktCABD* operon) along with an activator protein (LktC) responsible for post-translational acylation, which is essential for full cytolytic activity [17].

### 2.3. Bordetella Spp.

*Bordetella pertussis*, the causative agent of whooping cough, produces CyaA, a bifunctional RTX toxin that comprises an N-terminal adenylate cyclase domain with C-terminal RTX repeats. Upon binding to host phagocytes, CyaA translocates its catalytic domain into the cytoplasm, where it dramatically elevates intracellular cAMP levels, that can impair immune cell function. In addition to this enzymatic activity, CyaA exhibits pore-forming cytolytic activity. CyaA requires post-translational acylation by the CyaC enzyme for full activity [9,18]. Secretion of CyaA is mediated by a dedicated T1SS encoded by the *cyaCABD* operon, comprising CyaB, CyaD, and TolC-like protein that provides the outer membrane channel [19]. Unlike typical RTX cytolysins that primarily disrupt membranes, CyaA also alters host cell signaling through adenylate cyclase activity [20].

### 2.4. Vibrio and Shewanella

*Vibrio cholerae* secretes a massive multifunctional RTX toxin known as MARTX (Multifunctional-Autoprocessing RTX), encoded by *rtxA*. This toxin exemplifies an expanded RTX architecture, integrating multiple effectors into a single polypeptide for delivery into host cells [21]. Upon translocation, MARTX auto-releases effectors—including an actin cross-linking domain—via its internal cysteine protease domain (CPD), which responds to host inositol hexakisphosphate [22]. This autoprocessing adds a layer of post-translational regulation. One consequence of this activity is actin depolymerization and cell rounding, caused by covalent cross-linking of G-actin into non-polymerizable oligomers [23].

Other *Vibrio* spp. encode homologous MARTX toxins. *V. vulnificus* produces RtxA1, a MARTX-like toxin essential for cytotoxicity and virulence during septicemia, as shown by attenuated phenotypes in *rtxA1*-deficient strains [24]. *V. anguillarum*, a fish pathogen, expresses both *rtxA* and the hemolysin gene *vah1*, which are upregulated during infection, suggesting roles in virulence [25]. Related marine species also harbor RTX-like systems. *Shewanella* genomes encode conserved operons predicted to encode RTX toxins [26] and *Photobacterium damselae* produces Phobalysin, an RTX hemolysin implicated in fish pathogenesis and possibly in interbacterial competition [27].

### 2.5. RTX Toxins in Other Genera

*Kingella kingae*, a leading cause of pediatric osteomyelitis and endocarditis, produces the RTX toxin RtxA—a pore-forming cytolysin that targets leukocytes, synovial fibroblasts, and osteoblast-lineage cells—thereby contributing to joint and bone tissue damage during infection [28]. *Aggregatibacter actinomycetemcomitans*, an oral pathogen associated with endocarditis and periodontitis, secretes leukotoxin A (LtxA), an RTX family member that selectively targets human leukocytes and plays a central role in aggressive periodontal disease [29]. RTX toxins are broadly distributed among Gram-negative bacteria and exhibit functional specialization across different bacterial species. In *Moraxella bovis*, the RTX cytotoxin MbxA—encoded by the *mbxCABD* operon—is cytotoxic to bovine neutrophils and erythrocytes, contributing to infectious bovine keratoconjunctivitis [30]. Despite taxonomic and functional diversity, RTX toxins are typically controlled by pathogen-specific regulatory networks that are integrated with their ecological context and virulence strategies [6,15]. A comparative summary of key RTX toxins—including their secretion systems, host targets, and regulation—is presented in Table 1, illustrating both the architectural diversity and mechanistic convergence of these calcium-dependent toxins across pathogens.

## 3. Mechanisms of RTX Toxin Regulation

Effective regulation of RTX toxin expression is critical, as these large virulence factors are energetically expensive to produce and, if mis-expressed, can disrupt the bacterium’s own cellular integrity or environmental niche [15]. To prevent such detrimental effects, bacteria integrate transcriptional and post-transcriptional control with environmental sensing and coordination of secretion and activation [6]. Below, we describe these regulatory mechanisms across representative pathogens.

**Figure 2:**
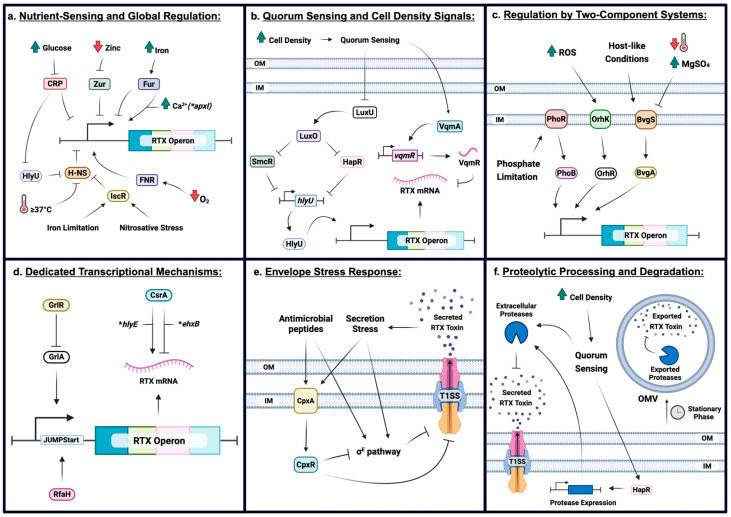
Multilayered regulation of RTX toxin expression in Gram-negative bacteria. RTX toxin expression is controlled by interconnected regulatory pathways that integrate environmental sensing, metabolic status, population density, and envelope homeostasis. (**a**) Global and nutrient-responsive regulators, including CRP, Fur, Zur, H-NS, FNR, IscR, and the anti-repressor HlyU, link RTX operon expression to carbon availability, metal homeostasis, oxygen tension, temperature, and nitrosative stress. (**b**) Quorum sensing and cell density-dependent regulation modulate RTX expression through the LuxO–LuxU phosphorelay and LuxR-type regulators (SmcR in *V. vulnificus* and HapR in *V. cholerae*), which repress *hlyU* and RTX transcription at high cell density, while LuxO-dependent small RNAs promote RTX expression at low cell density. (**c**) Two-component systems, including PhoBR, BvgAS, and OrhK/OrhR, regulate RTX expression in response to phosphate limitation, oxidative stress, and host-associated cues. (**d**) Dedicated transcriptional mechanisms such as RfaH-dependent antitermination and specific transcriptional regulators ensure efficient expression of long RTX-encoding operons. (**e**) Envelope stress responses (e.g., CpxAR and sigma E (σ^E^) pathways) fine-tune RTX expression and secretion in response to antimicrobial pressure and secretion stress. (**f**) Post-translational regulation, including quorum-dependent protease expression, extracellular degradation, and outer membrane vesicle (OMV) production, further modulate RTX toxin levels during stationary phase and high cell density. Created in BioRender. Dozois, C. (2026) https://BioRender.com/3jupd6x (accessed on 19 December 2025).

Overall, RTX toxin expression is governed by multilayered regulatory networks that integrate transcriptional repression and activation, metabolic and nutrient sensing, quorum-dependent signaling, envelope stress responses, and post-transcriptional control. These regulatory check points allow pathogens to suppress RTX production under non-infectious conditions while enabling rapid, context-dependent induction during host interaction. The major regulatory elements governing RTX toxin expression are summarized schematically in Figure 2.

In the sections below, we first outline regulation by DNA-binding transcriptional regulators and two-component systems, followed by post-transcriptional and post-translational mechanisms, and finally environmental and niche-specific cues that fine-tune RTX expression across diverse Gram-negative pathogens.

### 3.1. Transcriptional Regulation: Activators, Repressors, and Two-Component Systems

RTX toxin gene expression is controlled at the transcriptional level by global and local regulators that respond to host signals, stress cues, nutrient availability, and quorum density. The following sections detail the major regulatory systems controlling RTX encoding genes in diverse pathogens.

#### 3.1.1. Global Repressors and Anti-Repressors

H-NS-mediated silencing and HlyU anti-repression: The histone-like nucleoid structuring protein H-NS is a major global repressor of horizontally acquired genes, including RTX operons. It acts by binding AT-rich promoter regions to exclude RNA polymerase, trap transcriptional initiation complexes, or induce DNA looping [31,32]. In *V. cholerae*, RNA-seq analysis of a Δ*hns* mutant revealed marked upregulation of the RTX-encoding locus. The *rtxCA* and *rtxBDE* operons were expressed 4.7- and 2.5-fold higher, respectively, than in the wild-type strain, confirming H-NS as a repressor of genes encoding both the toxin and its secretion machinery [33]. Similarly, in *V. vulnificus*, *rtxC* and *rtxA1* were overexpressed 11.2- and 15.9-fold, respectively, in *hns* mutants, reinforcing the repressive role of H-NS controlling regulation of RTX-encoding genes [34]. Temperature modulates H-NS DNA-binding affinity, and at a higher temperatures, such as 37 °C and above in some species, is reduced and facilitates RTX expression under host physiological conditions [35].

HlyU, a member of the ArsR/SmtB transcription factor family [36,37], functions as an anti-repressor by competing with H-NS for promoter binding. In *V. vulnificus*, HlyU binds to two upstream sites that overlap with H-NS binding regions within the *rtxA1* promoter. It exhibits higher DNA-binding affinity than H-NS and displaces it, relieving repression without directly activating transcription [34,37]. HlyU also activates the *vvhBA* operon in *V. vulnificus* by binding upstream of the *vvhA* promoter [37,38]. It antagonizes H-NS and works in conjunction with the stress-responsive regulator IscR to relieve HN-S mediated repression in response to host-derived cues such as nitrosative stress and iron limitation. This regulatory coordination enables precise cytolysin induction during infection [38].

Together, the interplay between H-NS silencing and HlyU-mediated anti-repression ensures that RTX toxins and associated virulence factors are regulated and expressed only during specific stages of host colonization or in response to defined environmental conditions.

#### 3.1.2. Nutrient-Sensing and Global Regulation

Iron regulation via Fur: The ferric uptake regulator (Fur) links iron availability to RTX toxin expression across multiple Gram-negative pathogens. In *V. vulnificus*, Fur represses *rtxA1* expression under iron-replete conditions, thereby preventing host cell lysis and conserving bacterial resources under iron replete conditions [39]. Similarly, in *A. pleuropneumoniae*, *apxI* expression is both iron- and calcium-inducible, with Fur acting as a dual-function regulator whose activity is modulated by calcium availability [40]. In *V. cholerae*, Fur directly represses *hlyA* by binding to a promoter region spanning −164 to −75 bp—partially overlapping the HlyU binding site—suggesting competitive exclusion of this activator and a hierarchical regulatory mechanism. In *V. vulnificus*, Fur also represses the *vvhBA* operon by binding near the transcription start site (−32 to +2 bp), where it directly interferes with RNA polymerase recruitment, independently of HlyU [37]. Overall, iron homeostasis and Fur play a central role as an iron-sensitive transcriptional repressor, coordinating RTX expression with iron limitation during host colonization and infection.

Notably, Fur displays divergent regulatory roles across RTX-producing pathogens, functioning as a transcriptional repressor in some species while acting as an activator in others. This differential regulation highlights mechanistic differences that may reflect variation in promoter architecture, co-regulator interactions, or experimental conditions.

Carbon catabolite repression via cAMP-CRP: Carbon availability modulates RTX gene expression through the cAMP receptor protein (CRP). In *V. cholerae*, CRP binds upstream of the *rtxBDE* operon and represses transcription, linking toxin expression to central metabolic status [41]. In *V. vulnificus*, CRP directly represses *rtxA1* expression by binding to specific sites within the upstream promoter region [42]. Lee, Hwang, Choi, Jang, Lee, Chung, Kim and Choi [6] further showed that CRP represses *rtxA1* transcription in *V. vulnificus*, and that this repression is relieved by exogenous glucose. CRP binds directly and specifically to the upstream region of the *rtxA1* promoter and also binds upstream of *hlyU*, repressing expression of both of these transcripts. Since *hlyU* is a known activator of *rtxA1*, this dual regulation adds an additional layer of control over RTX gene expression. In *A. actinomycetemcomitans*, leukotoxin production is abruptly halted by a fructose pulse, underscoring the strong link between fermentable carbon availability and toxin regulation [43]. Together, these findings emphasize the effect of carbon metabolism and CRP- mediated catabolite repression on regulation of RTX toxins.

#### 3.1.3. Quorum Sensing and Cell Density Signals

In *Vibrio* spp., quorum sensing (QS) pathways regulate RTX toxin production through hierarchical control of global regulators. In *V. vulnificus*, quorum-sensing is tied to a TCS cascade (LuxO/LuxU) that ultimately controls SmcR (LuxR-type regulator); while not a simple TCS output, this quorum pathway intersects with RTX regulation by controlling HlyU levels. In *Vibrio vulnificus*, the LuxO–LuxU two-component system represses SmcR, a LuxR-type QS master regulator, which indirectly represses RTX expression by binding upstream of *hlyU* and downregulating its transcription at high cell density [37,44]. Similarly, in *V. cholerae*, HapR—a homolog of SmcR—directly represses *hlyA* transcription by binding to its promoter region [37,45]. Both HapR and SmcR can converge on *hlyU*, a central activator of RTX genes, indicating that QS circuits modulate exotoxin expression by downregulating *hlyU* during late infection stages. This population-dependent repression helps conserve energy and prevents excessive host tissue damage once colonization is established. Additionally, *vqmR*, a QS-regulated small RNA, represses *rtxHCA* expression in *V. cholerae*, highlighting a multilayered QS-mediated control of RTX genes across different species [37].

#### 3.1.4. Regulation by Two-Component Systems (TCS)

Many RTX producing bacteria use TCS signaling to adjust toxin production in response to environmental stimuli.

The BvgAS system in *Bordetella*: The BvgS-BvgA two-component system serves as the master regulator of virulence in *Bordetella* species, including control of *cyaA* expression. Under host-like conditions, the sensor kinase BvgS phosphorylates the response regulator BvgA, which then binds upstream of *cyaA* to activate its transcription to induce high-level toxin expression. This system operates by default in an “on” state, promoting *cyaA* expression. However, environmental signals such as low temperature or the presence of magnesium sulfate (MgSO_4_) shift the system to an “off” state, reducing BvgA phosphorylation and repressing *cyaA* transcription. If BvgS is inactivated by modulators (sulfate, low temp), BvgA~P levels drop and *cyaA* transcription ceases, ensuring the adenylate cyclase toxin is not produced outside the host [46].

Oxidative stress-responsive controlled TCS regulation in *E. coli*: In extra-intestinal pathogenic *E. coli* strains such as some uropathogenic *E. coli*, the two-component system OrhK/OrhR (C3564/C3565), encoded on a pathogenicity island, responds to oxidative stress inside host macrophages. According to a proposed model, OrhK senses reactive oxygen species (ROS) and activates OrhR through phosphorylation. The phosphorylated form of OrhR is suggested to activate expression of the *c3566-c3568* genes that are adjacent to the *hlyCABD* operon(*c3569-c3572*), leading to increased production of hemolysin (HlyA). This hemolysin, once processed by HlyC and secreted via the HlyB/HlyD system, contributes to host cell pyroptosis. This pathway enables UPEC to adjust toxin expression in response to oxidative cues encountered during infection [47]. Disruption of OrhK or OrhR results in reduced hemolysin production, impaired inflammasome activation, and diminished macrophage cytotoxicity, highlighting the role of this TCS in coordinating oxidative stress adaptation with virulence [47].

The PhoB-PhoR TCS and phosphate limitation: PhoB–PhoR responds to cytoplasmic phosphate limitation and governs both phosphate scavenging and stress-related gene expression [48]. In *E. coli* O157:H7, a PhoB-regulated promoter upstream of the RTX operon has been identified, and phosphate starvation is required for expression [49]. In *Synechocystis* sp., phosphate limitation induces PhoB-dependent transcription of the gene encoding the RTX-like protein, which constitutes one of the most abundant proteins in the extracellular proteome under these conditions [50].

#### 3.1.5. Dedicated Transcriptional Mechanisms

RfaH-dependent transcription elongation: In *E. coli* strains, RfaH is required for wild-type expression of the *hlyCABD* operon and enhances transcript elongation, consistent with its role as a transcriptional anti-terminator that supports transcription of promoter-distal genes [51,52]. In *rfaH* mutants, both *hly* mRNA and HlyA protein levels are reduced, leading to diminished hemolytic activity. This regulation involves a conserved 39-bp JUMPStart sequence in the untranslated leader region of RfaH-responsive operons, which participates in the same functional pathway as RfaH to promote efficient gene expression. Modulation of toxin efficacy may also reflect RfaH-dependent changes in *E. coli* outer membrane chemotype, supporting the involvement of lipopolysaccharide in hemolysin activity [51].

Other transcriptional inputs: Beyond canonical regulators and two-component systems, RTX toxin expression is subject to modulation by global stress responses and virulence cross-regulation. In *Serratia marcescens*, the CpxAR envelope stress system is activated at 37 °C and directly represses expression of the secreted protease PrtA by binding upstream of its gene. This thermoregulated repression prevents excess production of surface-expressed virulence factors under host-like conditions [53]. While PrtA is not an RTX toxin, this CpxAR-dependent control mechanism may reflect a broader strategy among Gram-negative pathogens to fine-tune secretion-associated virulence.

In *E. coli*, regulators of unrelated virulence modules can influence RTX-related hemolysins. The GrlA-GrlR regulatory system of the LEE island modulates enterohemolysin (EhxA) expression in EHEC. Deletion of *grlR* significantly increases Ehx activity, while GrlA overexpression induces *ehxCABD* expression even in the absence of Ler, establishing GrlA as a positive regulator of RTX hemolysin transcription [54]. Supporting its clinical importance, EhxA—an RTX-family pore-forming cytolysin—is frequently associated with hemorrhagic colitis and hemolytic uremic syndrome in EHEC infections and serves as a diagnostic marker of virulence [12].

Moreover, the post-transcriptional regulator CsrA acts as a dual modulator of hemolysin production. In EHEC O157:H7, CsrA represses plasmid-borne EhxA secretion by binding to the 5′ UTR of *ehxB*, thereby blocking ribosome access or promoting mRNA decay. Conversely, CsrA activates chromosomal *hlyE* expression by stabilizing its transcript through interaction with the 5′ leader sequence [55]. These findings highlight how envelope stress and carbon sensing pathways converge to modulate RTX expression via both transcriptional and post-transcriptional mechanisms.

Collectively, these insights support a model in which control of the RTX-encoding operons is embedded within multilayered regulatory networks that dynamically integrate envelope integrity, temperature, and metabolic state to mediate toxin production based on specific environmental cues.

### 3.2. Post-Transcriptional and Post-Translational Controls

Beyond transcription initiation, RTX toxin levels are further determined by regulatory inputs at the post-transcriptional and post-translational stages. These mechanisms ensure that toxin activity is spatially and temporally restricted to optimize pathogen fitness and minimize disfavorable production under less appropriate conditions.

#### 3.2.1. mRNA Stability and Translational Control

Small regulatory RNAs (sRNAs) have emerged as important modulators of toxin gene expression in bacteria. These molecules are classified into two main groups: *cis*-encoded sRNAs, also known as antisense RNAs, and *trans*-acting sRNAs. The latter group is often compared to eukaryotic microRNAs in both function and size [56].

In *V. cholerae*, the quorum-sensing regulator VqmA activates transcription of the regulatory RNA VqmR, which post-transcriptionally controls expression of multiple target mRNAs, including *rtx* toxin genes [57]. This finding highlights the contribution of *trans*-acting sRNAs to the post-transcriptional regulation of RTX toxins, linking quorum sensing to toxin modulation.

Although *cis*-encoded sRNAs have not yet been directly associated with RTX gene regulation, they play a significant role in other toxin systems and may similarly influence RTX-encoding operons through antisense interactions [56]. These insights emphasize the potential of sRNA-mediated regulation in mediating RTX expression at the post-transcriptional level.

#### 3.2.2. Enzymatic Activation: Acylation

RTX cytolysins are synthesized as inactive protoxins and require post-translational fatty acyl modification to become biologically active. This acylation occurs on the ε-amino groups of two internal conserved lysine residues and is mediated by the co-expressed toxin-activating acyltransferases such as HlyC, CyaC, or RtxC [58]. The acyltransferases select acyl chains of specific length from the bacterial pool of acyl–acyl carrier proteins (ACPs) and determine whether one or both lysine residues are modified. Functional studies revealed that the biological activity of RTX toxins depends on these acylation patterns: HlyA requires 14-carbon fatty acyl chains, and CyaA is activated exclusively by 16-carbon acyl chains [58].

In *K. kingae*, RtxA is activated by acylation on lysine residues K558 and/or K689 by RtxC, and the acylated form exhibits cytotoxic activity in a calcium-dependent manner [59]. For *B. pertussis* CyaA and *E. coli* HlyA, conserved tryptophan residues in the acylated segments are critical for host cell membrane penetration in the absence of β2 integrin receptors, suggesting that acylation supports a specific membrane-inserting conformation [58]. Collectively, these findings demonstrate that RTX toxin activation depends on acyl chain length, acylation position, and the conformational dynamics that enable cytotoxic activity.

#### 3.2.3. Secretion: Coupling to Translation and Environmental Feedback

RTX toxins are secreted via the T1SS, comprising an inner membrane ABC transporter (RtxB), a membrane fusion protein (RtxD), and an outer membrane channel (TolC). This process begins co-translationally—a phenomenon known as transertion—and requires substrate recognition of an unprocessed C-terminal secretion signal [60]. However, RTX export can itself be growth-phase regulated. In *V. cholerae*, stationary-phase cultures downregulate transcription of *rtxBDE*, resulting in intracellular accumulation of toxin and loss of extracellular activity. RTX toxins may also be released as components of outer membrane vesicles (OMVs), particularly during stationary phase. In *V. cholerae*, RTX was found associated with OMVs, suggesting the temporal regulation of extracellular toxin activity via packaging rather than direct secretion [61].

Building on the transcriptional regulatory networks described above, envelope stress responses (ESRs) also play an important role in regulating RTX toxin production at the level of secretion and envelope homeostasis. ESRs are essential for maintaining bacterial envelope integrity and are triggered by environmental perturbations [62]. Antimicrobial peptides, including polymyxin B and melittin, can activate ESRs such as CpxAR and sigma E (σ^E^) pathways in *E. coli* [63]. Activation of CpxAR contributes to tolerance against these peptides, suggesting that envelope stress signals are detected and transduced to adjust bacterial responses. In *Salmonella*, CpxAR activation also inhibits the σ^E^ pathway, highlighting interplay between distinct ESRs [64]. These findings support the idea that secretion stress may activate ESRs, including CpxAR and σ^E^, which in turn can repress secretion systems as a protective feedback mechanism.

Consistent with its broader role in envelope stress sensing, the CpxAR envelope stress response in uropathogenic *E. coli* (UPEC) also plays a direct role in fine-tuning HlyA levels during bladder colonization. During infection, UPEC downregulates *hlyA* expression in the bladder to minimize epithelial exfoliation and inflammation, allowing more stable colonization. Loss of CpxR leads to abnormally high HlyA production in vivo, resulting in excessive exfoliation and impaired bladder colonization efficiency. This establishes CpxR as a key regulator that maintains hemolysin expression within a range that promotes persistence rather than acute damage. These findings highlight that envelope stress pathways not only protect the bacterial envelope but also calibrate RTX toxin levels to optimize host-niche adaptation [65].

#### 3.2.4. Proteolytic Processing and Degradation

Certain RTX toxins are modified after they enter host cells. For example, the RTX toxin from *V. cholerae* can be destroyed by exported proteases, especially during late stages of growth when secretion is reduced and the toxin is associated with outer membrane vesicles [61]. These proteases limit toxin release in the environment and may contribute to controlling the timing or more gradual release of toxin over time.

In *Pseudomonas aeruginosa*, extracellular proteases such as LasB, LasA, and Protease IV (PIV) are secreted and become active in a cascade that is controlled by quorum sensing. Activation depends on removal of inhibitory propeptides, and the sequence of activation begins with LasB, which then activates PIV, followed by LasA [66]. Human elastase can also participate in this activation. These findings suggest that extracellular proteases can be regulated at both transcriptional and protein levels, depending on bacterial population density.

Although direct evidence of such a cascade for RTX toxins in *P. aeruginosa* is lacking, these findings suggest a general mechanism where QS controls extracellular protease activity, that may also potentially affect toxin stability and biological activity. Similarly, in *V. cholerae*, the HapR regulator which is produced at high cell density as part of the QS pathway can reduce toxin levels as bacterial numbers increase. HapR functions by blocking expression of *hlyA* by transcriptional repression and by promoting degradation of the toxin by increasing levels of the HapA metalloprotease. This process which is more readily observed on solid medium demonstrates that in late growth phase or high cell density *V. cholerae* reduces RTX toxin gene expression and production [45,67].

Together, these findings show that different bacteria use population-based signals in different ways to control toxin levels or promote their degradation. Some delay production until the population reaches a critical mass, while others use more complex systems to turn off toxin genes when they may no longer be needed.

#### 3.2.5. Protein Folding and Role of Chaperones

RTX toxins, unlike many exported proteins, do not pass through the periplasm and are secreted directly by a type I secretion system (T1SS). Still, cytoplasmic chaperones such as DnaK and DnaJ may influence their solubility prior to secretion. Studies on recombinant protein expression in *E. coli* have shown that DnaK/DnaJ can improve protein solubility but also enhance degradation via proteases like Lon and ClpP. These chaperones assist in refolding misfolded proteins but may also direct aberrant proteins toward degradation, thereby reducing overall yield. However, in expression systems lacking Lon and ClpP proteases—such as insect cells—DnaK/DnaJ enhanced both solubility and stability of recombinant proteins, supporting their folding role when uncoupled from proteolysis [68]. While RTX toxins have not been directly studied in this context, their large size and aggregation-prone nature suggest a potential dependence on general folding chaperones.

Together, these observations suggest that RTX toxins may transiently associate with cytoplasmic chaperones like DnaK/DnaJ to remain soluble prior to export. Folding likely occurs extracellularly, and excessive chaperone engagement may trigger degradation, underscoring the need for precise regulation during toxin biosynthesis and secretion.

### 3.3. Environmental and Host-Derived Cues Influencing RTX Regulation

RTX toxin expression is modulated by environmental stimuli and host-derived signals. These inputs act as contextual triggers that allow pathogens to confine toxin production to specific niches—typically host tissues—thereby minimizing metabolic burden and reducing the risk of premature immune activation. The principal regulatory signals and associated mechanisms that have been reported are summarized below.

#### 3.3.1. Temperature-Dependent Regulation

Temperature plays a central role in modulating RTX toxin gene expression across diverse Gram-negative pathogens. In *V. vulnificus*, the RTX-like gene *vva0331* exhibits significantly elevated transcription at lower environmental temperatures, with expression levels 4.7-fold and 2.3-fold higher at 30 °C and 20 °C, respectively, compared to 37 °C. Importantly, RTX protein secretion was only detected under these cooler conditions, suggesting that environmental temperatures favor both expression and export of the toxin [39]. Similar trends have been reported in *V. coralliilyticus*, where RTX expression was increased at 27 °C relative to 24 °C, correlating with the temperature range that triggers coral bleaching and disease. This elevated expression coincided with the upregulation of additional virulence factors such as hemolysins and flagellar proteins, indicating a coordinated response to warming seawater that enhances pathogenic potential [69]. In *Nautella italica*, RTX expression was also higher at 24 °C compared to 25 °C, although the biological implications of this narrow temperature preference remain uncertain [70].

In other aquatic animal pathogens such as *V. anguillarum*, the regulation of MARTX toxins also appears to be temperature-sensitive but in a distinct direction. Under iron-limited conditions, MARTX expression was 2.4-fold higher at 15 °C than at 25 °C, suggesting that low temperature may prime the bacterium for infection under nutrient stress typical of colder aquatic environments [25]. These collective findings underscore the plasticity of RTX toxin regulation in response to temperature shifts and suggest that each pathogen has evolved to fine-tune toxin expression according to its specific ecological niche and host environment. However, reported temperature-dependent effects on RTX expression vary substantially among species and experimental systems, and in some cases may reflect indirect effects on growth dynamics, secretion efficiency, or regulatory network crosstalk rather than direct temperature sensing mechanisms.

#### 3.3.2. Iron and Metal Availability

The expression of RTX toxins is influenced by iron and other metal ions through regulatory proteins such as Fur and Zur. In *A. pleuropneumoniae*, Hsu, Chin, Chang and Chang [40] showed that both iron and calcium increased the expression of the *apxICABD* operon encoding the ApxI RTX toxin. The Fur protein acts as a positive regulator in this system, especially in the presence of calcium. This activation appears to occur even without a typical Fur box in the upstream DNA, suggesting alternative binding features or indirect regulation.

In *V. vulnificus*, Chou, Peng, Yang, Kuo and Chang [39] found that Fur represses *rtxA1* expression under high iron conditions, and that this repression is lifted when iron availability becomes limited, such as during host infection. This indicates that Fur can downregulate RTX toxin gene expression when iron is abundant, and avoid unnecessary production particularly in environments outside of animal hosts.

Zinc also plays a regulatory role. Velasco, et al. [71] reported that in UPEC, the zinc-responsive regulator Zur binds to the promoter region of the *hlyII* operon and represses *hlyA* expression. Under zinc-limiting conditions, Zur does not bind, allowing increased toxin gene expression. Interestingly, the closely related *hlyI* operon was not affected by zinc or Zur, indicating that even within the same strain, different RTX operons may respond differently to regulatory cues such as metal availability.

In *V. cholerae*, Fur also regulates hemolysin encoding genes, including *hlyA*, where its presence leads to reduced expression during iron sufficiency [41]. Altogether, these findings highlight the importance of iron and zinc as environmental and host-derived signals that bacteria use to fine-tune RTX toxin gene expression depending on the surrounding environment.

#### 3.3.3. Oxygen Tension and Redox State

Low oxygen availability acts as a signal for RTX toxin induction in many Gram-negative pathogens. In *E. coli*, oxygen limitation enhances *hlyA* and *hlyD* expression via the fumarate and nitrate reduction regulator (FNR), a global transcription factor that is activated under anaerobic conditions. FNR becomes functional upon acquisition of a [4Fe–4S]^2+^ cluster in low O_2_, enabling DNA binding and transcriptional activation. Deletion of *fnr* significantly reduces *hlyA* and *hlyD* expression under anaerobic conditions but has no effect aerobically, confirming FNR’s role in hypoxia-responsive regulation [72]. A similar oxygen-dependent induction is observed in *A. actinomycetemcomitans*, where LtxA production increases 3- to 4-fold under anaerobic growth [73,74]. However, this effect varies by strain and suggests the involvement of additional regulatory inputs or FNR-like systems [75].

In *A. pleuropneumoniae*, all virulent serovars or strains produce both the ApxI and ApxII toxins, and high virulence requires the combined action of these two cytolysins [76]. Expression of ApxI and ApxII increases during late exponential or early stationary phase, when cell density is high or growth rate is reduced [77]. Furthermore, ApxI production is transcriptionally regulated by free Ca^2+^ ions, linking toxin expression to extracellular calcium availability [78]. In contrast, oxygen limitation does not significantly alter overall Apx toxin production compared to normoxic conditions [79]. Iron-responsive regulation further contributes to control of ApxI expression, as Fur enhances *apxICABD* transcription in the presence of calcium, while its regulatory effects vary under low calcium or iron conditions. In addition, a point mutation upstream of *apxIC* modulates expression in a calcium-, iron-, and Fur-dependent manner [40]. Collectively, these findings indicate that Apx toxin regulation collectively is dependent on growth phase, calcium availability, and iron-responsive regulatory inputs to fine-tune RTX toxin production during infection.

### 3.4. Niche-Specific and Host-Microbiome Contexts

RTX toxin regulation is shaped not only by broad environmental cues but also by the anatomical niche and microbial context in which the pathogen resides. This regulation is embedded within global virulence strategies and responds to both host-derived stimuli and interbacterial interactions. Many of these host-associated signals converge on regulatory systems discussed above, including envelope stress responses, quorum sensing circuits, and metabolic regulators, thereby linking environmental sensing to niche-specific RTX expression. From the gut to the bloodstream, RTX expression is fine-tuned to maximize pathogenic fitness while minimizing unnecessary metabolic burden or immune activation.

For example, bacterial contact with host cells or tissues can increase RTX toxin expression in some bacteria. In *V. vulnificus*, the RtxA1 toxin causes strong cell damage only when the bacteria are in close contact with host cells. This contact increases the expression and release of RtxA1 and its transporter, *rtxB1*. When the *rpoS* gene is mutated, this increase becomes weaker and slower. These findings show that host contact somehow elicits expression of RTX toxin genes, and RpoS plays an important role in this process [80].

#### 3.4.1. Intestinal Niches: Gut-Specific Signals and Microbiota Interactions

Inside the gut, bacteria such as *E. coli* and *V. cholerae* face a mix of host factors, including bile salts, limited oxygen, and the presence of other competing microbes. *V. cholerae* uses its MARTX toxin during intestinal infection to disrupt the gut lining, which promotes bacterial colonization and suppresses the host inflammatory responses [81]. In *V. vulnificus*, a related MARTX toxin with multiple domains causes damage and triggers signals inside gut cells, but some of these signals are blocked by other parts of the same toxin [82]. This fine-tuning allows the bacteria to cause disease and modulate the host innate response.

The low oxygen level in the gut is an important regulatory cue. In *E. coli*, for example, regulators like FNR and ArcA are activated when oxygen is low. These regulators work together to switch on or off specific genes when bacteria adapt to growth conditions in the intestine [83]. These regulators can also play a role in activation of RTX systems such as HlyA in *E. coli* [72].

#### 3.4.2. Respiratory Niches: BvgAS, Inflammation, and Redox Signals

In the respiratory tract, RTX toxins are expressed in response to environmental cues associated with infection and inflammation. *B. pertussis*, for example, produces the RTX adenylate cyclase toxin CyaA in greater amounts when exposed to serum proteins such as albumin and extracellular calcium—components commonly found in the inflamed airway. These host-derived factors trigger a notable increase in toxin secretion into the extracellular space, even without changes in gene transcription, suggesting that post-transcriptional mechanisms or secretion systems are responsive to environmental conditions and cues within the host respiratory tract [46].

In *M. haemolytica*, LktA is released in large quantities during acute lung infections. Studies of bovine pneumonia indicate that LktA expression is associated with the damaged, inflamed tissue environment. Hemorrhagic lesions provide both reduced oxygen and excess nutrients such as iron, creating a niche that supports LktA activity and persistence during disease progression [84].

Reactive oxygen species (ROS), particularly hydrogen peroxide produced by neutrophils, are another signal that can influence toxin gene expression. While direct RTX regulation by ROS remains to be fully elucidated, redox-active molecules are known to activate regulators like OxyR, which senses oxidative stress and adjusts bacterial gene expression accordingly. Evidence from other Gram-negative pathogens, including studies of protein thiol oxidation and redox transcription factors, suggests that RTX producing bacteria could exploit similar redox-sensitive pathways to fine-tune levels of toxin expression within the respiratory tract [85].

#### 3.4.3. Bloodstream and Deep Tissue Environments

After entering the bloodstream or internal tissues, pathogens like *V. vulnificus* and extra-intestinal pathogenic *E. coli* face nutrient limitation and immune stress. In *V. vulnificus*, the regulator HlyU plays a key role in activating the RTX toxin gene *rtxA1* as well as the *vvhA* gene which encodes an unrelated pore-forming toxin during early stages of infection [34,44]. As the bacterial population increases, the QS protein SmcR becomes active and suppresses *hlyU*, leading to reduced expression of these toxins [44]. This switch may help the pathogen transition from aggressive invasion to persistence.

In *E. coli*, *hlyA* promotes lysis of host cells like macrophages [86]. While the full mechanism is still being investigated, this activity may help the bacteria avoid immune clearance. Under iron-limited conditions, which are typical in host tissues, Fur repression is lifted. This allows the simultaneous expression of iron acquisition systems and genes encoding HlyA production and transport to increase toxin production to promote cell damage and/or impair host cellular immune defenses [80,87].

#### 3.4.4. Mucosal and Biofilm-Associated Niches

In the mouth, *A. actinomycetemcomitans* builds strong biofilms that help it survive and cause disease. It produces fimbriae, polysaccharides, and extracellular DNA to attach to surfaces and form a sticky matrix from protection. One key component is a sugar-based polymer called PGA, which is made by the genes in the *pgaABCD* operon. Inside this biofilm, the bacteria can hide from the immune system, partly because the biofilm matrix blocks some of the host immune defenses [88].

*A. actinomycetemcomitans* also produces the RTX toxin LtxA, which targets white blood cells. This helps abate immune cellular innate defenses during infection. The level of LtxA produced by different strains is variable and some strains produce much more LtxA than others due to changes in the promoter sequence [89].

Inside the biofilm, bacteria can sense environmental changes and respond by turning certain genes on or off. For example, changes in iron, oxidants, or host molecules can affect gene expression [88]. These biofilm-specific signals may regulate toxin production, although the exact mechanisms underlying regulation of LtxA are still not fully elucidated.

#### 3.4.5. Coordination with Other Virulence Determinants

HlyU, previously described as a key activator of RTX and other toxins in *V. vulnificus* can be pharmacologically inhibited by the small molecule CM14, which blocks its DNA-binding capacity. This lowers HlyU activity and expression of specific proteins and resulted in increased protection of mice by reducing body damage and inflammation [90]. Several regulators work alongside HlyU in these bacteria, including CRP, RpoS, Fur, ToxRS, AphB, and LeuO, as outlined in regulatory summaries of *V. vulnificus* virulence control [91].

In *B. pertussis*, the RisA regulator functions at times when the major BvgAS regulatory system is not active. RisA controls many genes including those involved in iron use, motility, and potentially other genes [92]. In addition, BvgA, a key regulator of the CyaA toxin, also activates other regulators and catabolic pathways, including those involved in carbohydrate and lipid metabolism and secretion systems, highlighting the close integration of virulence and metabolism to promote bacterial persistence in host niches [93].

A succinct summary of the major regulatory systems controlling RTX toxin expression and their general effects is provided in Table 2 to facilitate cross-species comparison and reader orientation.

Despite extensive descriptive studies, key questions remain unresolved, including how multiple regulatory layers are hierarchically integrated in vivo and to what extent in vitro observations accurately reflect regulatory dynamics that occur during infection.

## 4. Conclusions

RTX toxins represent a potent and diverse arsenal of toxic proteins employed by Gram-negative bacteria, with their regulation finely tuned to environmental, metabolic, and host-specific signals. Genes encoding RTX toxins are regulated at multiple levels, including transcriptional control by global regulators, niche-specific cues, quorum sensing, growth phase, redox stress, and post-transcriptional mechanisms. Regulatory mechanisms can vary by species and context, but common themes emerge: stringent repression under non-infectious conditions, rapid induction upon host contact, and integration of RTX expression within broader virulence and metabolic networks. Emerging tools in spatial transcriptomics, sRNA profiling, and synthetic biology promise to deepen our understanding of these complex systems. Targeting RTX regulatory pathways offers exciting potential for anti-virulence therapies to disarm pathogens without promoting development of resistance, positioning RTX regulation as both a model of bacterial control and a blueprint for novel interventions. For example, pharmacological inhibition of key regulators such as HlyU has already demonstrated protective effects in vivo, highlighting the feasibility of targeting RTX regulatory nodes rather than the toxins themselves. In parallel, emerging synthetic biology approaches, including CRISPRi-based repression of RTX operons or their regulators, offer powerful tools to selectively modulate toxin expression with minimal impact on bacterial viability. Together, these strategies illustrate how mechanistic insights into RTX regulation can be translated into innovative anti-virulence interventions.

Across diverse Gram-negative pathogens, several unifying principles of RTX regulation emerge. RTX genes are typically silenced under environmental or commensal conditions and are induced only upon exposure to host-associated cues such as temperature shifts, nutrient limitation, redox stress, or direct host cell contact. Regulatory integration with metabolic state, quorum sensing, and envelope stress responses ensures that RTX production is coordinated with bacterial fitness and broader virulence programs. These shared strategies highlight RTX regulation as an evolutionarily conserved feature in a variety of Gram-negative bacterial pathogens and underscore the potential of targeting regulatory nodes or networks rather than toxins themselves for anti-virulence intervention.

## Figures and Tables

**Table 1:** Overview of representative RTX toxins across several key pathogenic bacterial species.

Toxin	Producing Species	Host Target	Key Regulator(s)	Secretion/Activation Genes
HlyA	*E. coli*	RBCs, macrophages	H-NS, RfaH, CRP	*hlyCABD*, *hlyC*
CyaA	*B. pertussis*	Neutrophils	BvgAS	*cyaCABD*, *cyaC*
RtxA1	*V. vulnificus*	Epithelial cells	HlyU, Fur, CRP, SmcR	*rtxHCA*, *rtxBDE*
LktA	*M. haemolytica*	Bovine leukocytes	Possibly Fur, others unknown	*lktCABD*, *lktC*
MARTX	*V. cholerae*	Host cytoskeleton, actin	HlyU, HapR	*rtxA*, *rtxBDE*
ApxI-IV	*A.* *pleuropneumoniae*	Porcine epithelial & immune cells	OxyR, Fur	*apxI–IV*, *apxCABD*
LtxA	*A. actinomycetemcomitans*	Human leukocytes	Unknown (possibly H-NS)	*ltxCABD*, *ltxC*
RtxA	*K. kingae*	Bone/joint tissue	Unknown	*rtxCABD*

**Table 2:** Major regulatory systems controlling RTX toxin expression across Gram-negative pathogens.

Regulatory Category	Key Regulators/ Systems	General Impact on RTX Expression	Primary Signals	Species
Global transcriptional repression and anti-repression	H-NS, HlyU-like anti-repressors	Silencing under non-host conditions; derepression during infection	Temperature, DNA topology, host contact	*Vibrio*, *E*. *coli*
Metal and nutrient sensing	Fur, Zur, calcium- responsive regulators	Repression or activation depending on metal availability	Iron, zinc, calcium	*Vibrio*, *E. coli*, *A*. *pleuropneumoniae*
Metabolic regulation	CRP, CsrA	Coordination of RTX expression with metabolic state and growth	Carbon availability, growth phase	*Vibrio*, *E*. *coli*
Quorum sensing	HapR, SmcR, LuxR-type regulators	Cell-density-dependent repression or modulation	Autoinducers, population density	*Vibrio*
Two-component systems	BvgAS, PhoBR, OrhK/OrhR	Conditional activation or repression in response to environment	Temperature, phosphate, oxidative stress	*Bordetella*, *E*. *coli*
Envelope stress responses	CpxAR, σ^E^	Fine-tuning of RTX secretion and toxin levels	Envelope stress, secretion burden	*E*. *coli*, *Salmonella*
Post-transcriptional regulation	sRNAs, CsrA	Modulation of mRNA stability and translation	Growth phase, quorum sensing	*Vibrio*, *E*. *coli*
Post-translational activation	RTX acyltransferases	Control of toxin activation and cytotoxic potency	Acyl-ACP availability	Multiple RTX-producing pathogens

## Data Availability

No new data were created or analyzed in this study.
